# Carvedilol Inhibits Angiotensin II-Induced Proliferation and Contraction in Hepatic Stellate Cells through the RhoA/Rho-Kinase Pathway

**DOI:** 10.1155/2019/7932046

**Published:** 2019-11-07

**Authors:** Ying Wu, Zhen Li, Sining Wang, Aiyuan Xiu, Chunqing Zhang

**Affiliations:** ^1^Department of Gastroenterology, Shandong Provincial Hospital Affiliated to Shandong University, Jinan, Shandong Province, China; ^2^Shandong Provincial Engineering and Technological Research Center for Liver Diseases Prevention and Control, Jinan, China; ^3^Department of Geriatric Gastroenterology, Shandong Provincial Hospital Affiliated to Shandong University, Jinan, Shandong Province, China

## Abstract

**Aim:**

Carvedilol is a nonselective beta-blocker used to reduce portal hypertension. This study investigated the effects and potential mechanisms of carvedilol in angiotensin II- (Ang II-) induced hepatic stellate cell (HSC) proliferation and contraction.

**Methods:**

The effect of carvedilol on HSC proliferation was measured by Cell Counting Kit-8 (CCK-8). Cell cycle progression and apoptosis in HSCs were determined by flow cytometry. A collagen gel assay was used to confirm HSC contraction. The extent of liver fibrosis in mice was evaluated by hematoxylin-eosin (H&E) and Sirius Red staining. Western blot analyses were performed to detect the expression of collagen I, collagen III, *α*-smooth muscle actin (*α*-SMA), Ang II type I receptor (AT1R), RhoA, Rho-kinase 2 (ROCK2), and others.

**Results:**

The results showed that carvedilol inhibited HSC proliferation and arrested the cell cycle at the G0/G1 phase in a dose-dependent manner. Carvedilol also modulated Bcl-2 family proteins and increased apoptosis in Ang II-treated HSCs. Furthermore, carvedilol inhibited HSC contraction induced by Ang II, an effect that was associated with AT1R-mediated RhoA/ROCK2 pathway interference. In addition, carvedilol reduced *α*-SMA expression and collagen deposition and attenuated liver fibrosis in carbon tetrachloride (CCl_4_)-treated mice. The *in vivo* data further confirmed that carvedilol inhibited the expression of angiotensin-converting enzyme (ACE), AT1R, RhoA, and ROCK2.

**Conclusions:**

The results indicated that carvedilol dose-dependently inhibited Ang II-induced HSC proliferation by impeding cell cycle progression, thus alleviating hepatic fibrosis. Furthermore, carvedilol could inhibit Ang II-induced HSC contraction by interfering with the AT1R-mediated RhoA/ROCK2 pathway.

## 1. Introduction

Liver fibrosis and cirrhosis are worldwide public health problems with severe complications including portal hypertension and hepatic failure [1]. They are chronic inflammation and tissue repair processes in which excess extracellular matrix (ECM) deposition occurs [2, 3]. Hepatic stellate cells (HSCs) are the main fibrogenic cell type in the space of Disse, and activated HSCs proliferate and secrete large amounts of ECM during fibrosis development. Moreover, HSC contraction can increase hepatic sinusoidal pressure, which is important in the development of portal hypertension [4–6].

Studies have suggested that the renin-angiotensin system (RAS) is important in the pathogenesis of liver fibrosis [7, 8]. Angiotensin II (Ang II) has been identified as the main effector molecule of the RAS and plays an important role in intrahepatic circulation regulation. Moreover, excess Ang II promotes the inflammatory response and liver fibrosis [9, 10]. Activation of the RAS resulting in Ang II type I receptor (AT1R) stimulation plays a crucial role in HSC activation and fibrogenesis [11]. AT1R is associated with the stimulation and activation of several signaling pathways involved in cell contraction and ECM production. The RhoA/Rho-kinase pathway is one of the pathways that participate in the development of hepatic fibrosis and portal hypertension [12, 13]. After activation of AT1R by Ang II, RhoA activates Rho-kinase, which increases myosin light chain (MLC) phosphorylation and related contraction [12]. In addition, Kitamura et al. [14] demonstrated that the Rho/Rho-kinase pathway is partly involved in the RAS and affects the processes of liver fibrosis and steatosis. Therefore, we infer that Ang II may activate the AT1R-mediated RhoA/Rho-kinase pathway to participate in the activation, proliferation, and contraction of HSCs.

Carvedilol is an adrenergic receptor blocker that can effectively reduce portal pressure and is used to prevent esophageal variceal bleeding. As a relatively new nonselective beta blocker (NSBB), carvedilol is more effective than propranolol at reducing portal hypertension in patients with cirrhosis [15]. In addition to directly reducing portal blood flow, carvedilol may exert the potential beneficial effect of decreasing vascular resistance in the liver by inhibiting HSC contraction and alleviating liver fibrosis. It has been reported that carvedilol treatment attenuates liver lesions [16]. Tian et al. [17] demonstrated that carvedilol can attenuate hepatic fibrosis by ameliorating oxidative stress in rats with bile duct ligation. The present study evaluated the effects of carvedilol on Ang II-induced HSC proliferation and contraction and further elucidated the underlying molecular mechanisms of its effects on liver fibrosis and portal hypertension.

## 2. Materials and Methods

### 2.1. Cell Culture

Human LX-2 HSCs were obtained from the ATCC (VA, United States) and cultured in DMEM (Invitrogen, New York, USA) supplemented with 10% fetal bovine serum (BI, Biological Industries, Beit Haemek, Israel) at 37°C with 5% CO_2_.

### 2.2. Cell Proliferation Assay

HSCs (3 × 10^3^ cells/well) were seeded in 96-well plates and cultured overnight in DMEM with 10% fetal bovine serum. The concentrations of carvedilol were determined according to our previous experiments [18]. After the cells were treated with Ang II and carvedilol at the indicated concentrations for 24 hours, 10 *μ*L of CCK-8 (Dojindo, Kumamoto, Japan) reagent was added to each well. The plates were incubated at 37°C for 1 hour, and spectro-photometric absorbance was measured at 450 nm using a scanning multiwell spectrophotometer (Bio-Rad Model 550, CA, USA). The results are based on triplicate experiments.

### 2.3. Cell Cycle Assay

HSCs were seeded in 6-well plates at a density of 5 × 10^4^ cells/well. The cells were cultured overnight in serum-free medium and then treated with Ang II and various concentrations of carvedilol for 24 hours. The cells were then collected and fixed in 70% ethanol overnight at 4°C. Cell cycle phases were detected with a Muse Cell Cycle Assay Kit (Merck-Millipore, Darmstadt, Germany) according to the manufacturer's protocol. The percentages of cells in each phase of the cell cycle were determined and analyzed with a Muse Cell Analyzer (Merck-Millipore, Darmstadt, Germany). The results are based on triplicate experiments.

### 2.4. Analysis of Apoptosis by Flow Cytometry

HSCs (5 × 10^4^ cells/well) were seeded in 6-well plates, cultured overnight, and then treated with Ang II and various concentrations of carvedilol for 24 hours. A PE Annexin V Apoptosis Detection Kit I (BD Biosciences, USA) was used for analysis according to the manufacturer's protocol. The percentages of apoptotic cells were determined by flow cytometry (FACSCalibur Flow Cytometer, BD Biosciences, USA). The data were analyzed with the FACSDiva 7.0 software. The results are based on triplicate experiments.

### 2.5. Analysis of Apoptosis by Fluorescence Staining

HSCs were seeded in 6-well plates at a density of 2 × 10^4^ cells/well and cultured overnight. After the cells were treated with Ang II and various concentrations of carvedilol for 24 hours, nuclear morphological changes and DNA fragmentation, indicating apoptotic cells, were detected with a Hoechst 33258 fluorescence staining kit (Beyotime, Shanghai, China) according to the manufacturer's protocol. Apoptotic HSCs were observed under a fluorescence microscope (Olympus, Tokyo, Japan).

### 2.6. Cell Contraction Assay

Hydrated collagen gels were prepared in 24-well plates using type I rat tail tendon collagen according to the manufacturer's protocol (Shengyou Biotechnology, Hangzhou, China). HSCs (3 × 10^4^ cells/well) were cultured on the collagen gels for 24 hours. Then, Ang II and various concentrations of carvedilol/ROCK inhibitor (Y-27632) were added into each well. The diameters of the collagen lattices were monitored until 24 hours after stimulant addition. The results are based on triplicate experiments.

### 2.7. Scanning Electron Microscopy (SEM)

Aseptic coverslips were placed in 6-well plates, and HSCs (5 × 10^4^ cells/well) were seeded in the plates and cultured for 24 hours. After the cells adhered to the coverslips, Ang II and different concentrations of carvedilol were added. When the cells contracted, the coverslips were washed with PBS and immobilized immediately in electron microscopy fixative (Servicebio, Wuhan, China) for 1 hour. After the coverslips were fixed, serially dehydrated, and dried, they were observed under a scanning electron microscope (SU8010, Hitachi, Japan).

### 2.8. Animal Model

Forty adult male C57BL/6 mice were purchased from the Experimental Animal Center of Shandong University (Jinan, China). The mice were kept at a constant temperature and given laboratory chow and water *ad libitum*. The experimental protocols were approved by the Animal Care and Utilization Committee of Shandong Provincial Hospital affiliated to Shandong University.

The experiment was performed according to our previous method for constructing a mouse model of liver fibrosis [19]. The liver fibrosis model was successfully constructed by intraperitoneal injection of CCl_4_ (25% CCl_4_ in olive oil) twice a week for 6 weeks, and mice in the carvedilol group were treated with carvedilol at the same time (10 mg/kg/d, by gavage). Mice in the control group were injected intraperitoneally with olive oil alone. The survival rate of the mice was 80% until the end of the experiment.

### 2.9. Histological Examination and Immunohistochemical Staining

Liver tissues were fixed with 4% formaldehyde and sectioned for H&E and Sirius Red staining. Liver fibrosis was assessed according to the METAVIR scale (F0, no liver fibrosis; F1, portal fibrosis; F2, periportal fibrosis; F3, bridging fibrosis; F4, liver cirrhosis) [20]. After being deparaffinized and serially dehydrated, the sections were treated with hydrogen peroxide for 30 minutes and then incubated with primary antibody overnight at 4°C. The primary antibodies used for immunohistochemical staining were anti-angiotensin-converting enzyme 1 (ACE1) antibody (1 : 200, Abcam, MA, USA) and anti-fibronectin antibody (1 : 2000, Abcam, MA, USA). After treatment with a biotinylated secondary antibody for 30 minutes at 37°C, the positive areas were stained with diaminobenzidine (DAB) and the nuclei were counterstained with hematoxylin. Immunohistochemical analysis was performed with Image-Pro Plus 6.0 software.

### 2.10. Western Blot Assay

Proteins were extracted for Western blot analysis, and concentrations were detected by the bicinchoninic acid (BCA) protein determination method. The proteins were separated by sodium dodecyl sulfate polyacrylamide gel electrophoresis (SDS-PAGE) and transferred to polyvinylidene fluoride (PVDF) membranes, which were incubated in 5% nonfat milk for 1 hour and then with primary antibodies overnight at 4°C. After secondary antibody incubation and membrane washing, the bands were detected using enhanced chemiluminescence (Millipore, USA). The results were analyzed with ImageJ software.

### 2.11. Statistical Analysis

All data are expressed as the mean ± standard deviation. Student's *t*-test or one-way ANOVA were used for statistical significance analysis. Values of *P* < 0.05 were considered statistically significant.

## 3. Results

**Figure 1:**
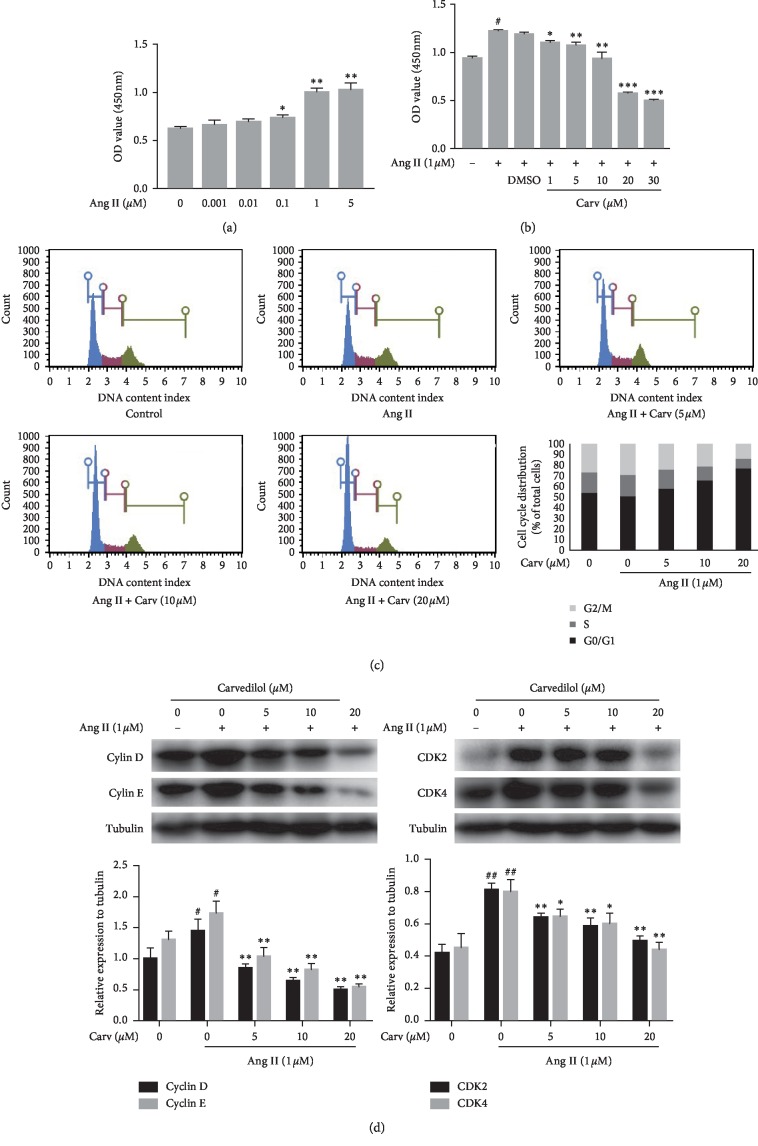
(a) HSCs were treated with different concentrations of Ang II and the proliferative effects were evaluated by CCK-8. ^*∗*^*P* < 0.05 versus control; ^*∗∗*^*P* < 0.01 versus control. (b) After HSCs were stimulated with Ang II at 1 *μ*M and treated with different concentrations of carvedilol, the proliferative effects were evaluated by CCK-8. ^#^*P* < 0.05 versus control; ^*∗*^*P* < 0.05 versus Ang II group; ^*∗∗*^*P* < 0.01 versus Ang II group; ^*∗∗∗*^*P* < 0.001 versus Ang II group; *P* > 0.05 (Ang II + DMSO) group versus Ang II group. (c) Effect of carvedilol on cell cycle progression in HSCs was detected by a muse cell analyzer. (d) Western blot analyses of cell cycle regulatory proteins. ^#^*P* < 0.05 versus control; ^##^*P* < 0.01 versus control; ^*∗*^*P* < 0.05 versus Ang II group; ^*∗∗*^*P* < 0.01 versus Ang II group. Representative blots were obtained from three independent experiments.

*3.1. Carvedilol Inhibited Proliferation and Cell Cycle Progression in HSCs Treated with Ang II*. The effect of Ang II on HSC proliferation was evaluated by the CCK-8 assay. The results showed that Ang II enhanced HSC proliferation in a dose-dependent manner and produced a significant effect at 1 *μ*M (Figure 1(a)). Carvedilol dose-dependently inhibited the proliferation of HSCs stimulated with Ang II. Moreover, there was no significant difference between the Ang II (1 *μ*M) group and the Ang II (1 *μ*M) + DMSO group (Figure 1(b)). To investigate the mechanism by which carvedilol reduced HSC proliferation, cell cycle analysis was performed in carvedilol-treated HSCs. The data showed that carvedilol dose-dependently prolonged the G0/G1 phase, which reduced the number of cells in the S and M phases among HSCs activated by Ang II (Figure 1(c), *P* < 0.05 vs. the control and Ang II groups). The results demonstrated that carvedilol might arrest cell cycle progression to inhibit HSC proliferation. Cyclins and cyclin-dependent kinases (CDKs) are known to play important regulatory roles in cell cycle progression. Cyclin D, cyclin E, CDK2, and CDK4 are crucial regulatory proteins of the G1 phase. The Western blot results showed that the expression of these proteins was increased in the Ang II group, while carvedilol notably inhibited this Ang II-mediated upregulation (Figure 1(d)). In general, our results show that carvedilol inhibited HSC proliferation and caused G0/G1 phase cell cycle arrest by altering cell cycle regulatory proteins in Ang II-treated HSCs.

### 3.2. Carvedilol Increased Apoptosis in HSCs Treated with Ang II

**Figure 2:**
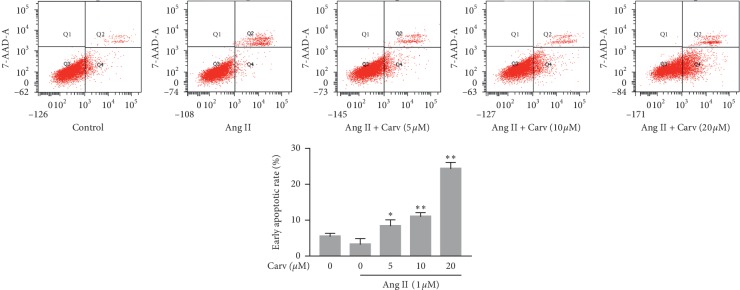
Flow cytometry analyses of apoptosis in HSCs treated with Ang II (1 *μ*M) and carvedilol for 24 hours. ^*∗*^*P* < 0.05 versus Ang II group; ^*∗∗*^*P* < 0.01 versus Ang II group. Results were obtained from triplicate experiments.

**Figure 3:**
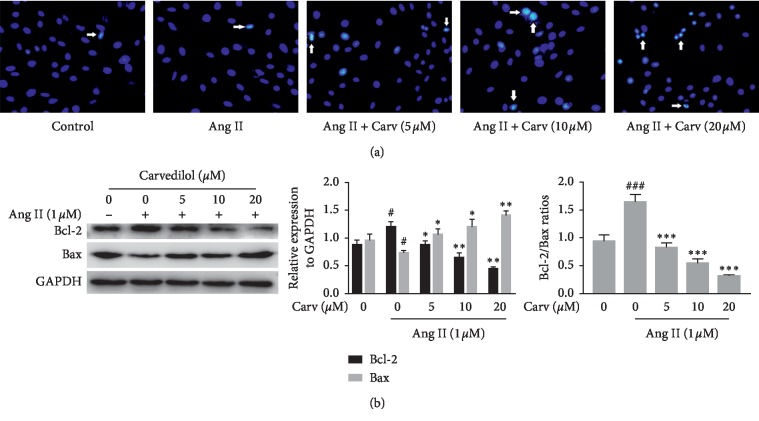
(a) Hoechst fluorescence staining of apoptosis in HSCs treated with Ang II (1 *μ*M) and carvedilol for 24 hours. The nuclei of apoptotic HSCs exhibit bright blue fluorescence, as indicated by white arrows. (b) Western blot analyses of Bcl-2 and Bax. ^#^*P* < 0.05 versus control group; ^###^*P* < 0.001 versus control group; ^*∗*^*P* < 0.05 versus Ang II group; ^*∗∗*^*P* < 0.01 versus Ang II group; ^*∗∗∗*^*P* < 0.001 versus Ang II group. Representative blots were obtained from three independent experiments.

As HSC apoptosis plays a key role in the reversal of liver fibrosis, we detected the apoptotic rate of HSCs by flow cytometry analysis. The results demonstrated that carvedilol dose-dependently enhanced the proportion of Ang II-treated HSCs in early apoptosis. The proportion of apoptotic HSCs was significantly increased by 20 *μ*M carvedilol treatment (Figure 2). When apoptosis occurs, nuclear fragmentation appears, and chromatin condensation becomes evident in cells. Hoechst staining showed that apoptotic HSCs exhibited nuclear fragmentation and DNA condensation with striking brilliant blue staining after treatment with carvedilol (Figure 3(a)). The regulation of apoptosis is extremely complicated, and Bcl-2 family proteins located in mitochondria play vital roles in the process. Western blot analysis revealed increased expression of the antiapoptotic protein Bcl-2 in the Ang II group; however, expression was downregulated by carvedilol in a dose-dependent manner. In contrast, expression of the proapoptotic protein Bax was decreased in the Ang II group and upregulated dose-dependently by carvedilol (Figure 3(b)). *In vitro* experimental results suggest that the mitochondrial apoptosis pathway is involved in the apoptosis induced by carvedilol.

### 3.3. Carvedilol Inhibited Ang II-Induced HSC Contraction

**Figure 4:**
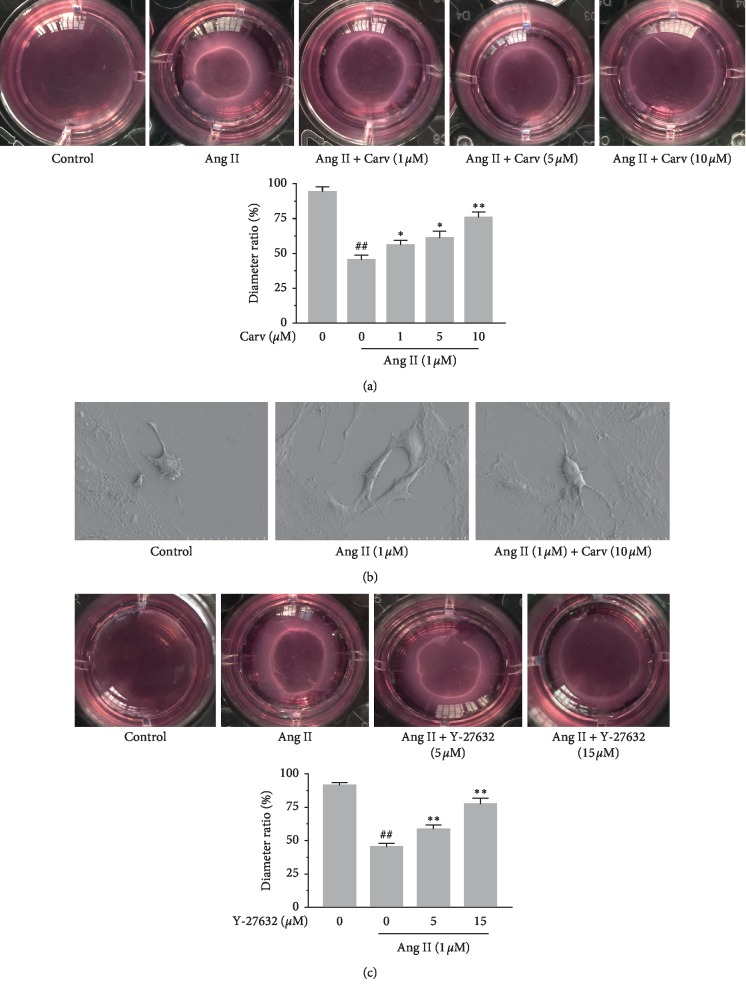
(a) Effect of carvedilol on HSC contraction was determined by collagen gel assay. HSCs were cultured on collagen lattices and treated with Ang II (1 *μ*M) and different concentrations of carvedilol. ^##^*P* < 0.01 versus control group; ^*∗*^*P* < 0.05 versus Ang II group; ^*∗∗*^*P* < 0.01 versus Ang II group. Results were obtained from triplicate experiments. (b) Morphological changes of HSCs were observed under SEM (magnification ×1000). (c) Effect of Y-27632 on HSC contraction was determined by collagen gel assay. HSCs were cultured on collagen lattices and treated with Ang II (1 *μ*M) and different concentrations of Y-27632. ^##^*P* < 0.01 versus control group; ^*∗∗*^*P* < 0.01 versus Ang II group. Results were obtained from triplicate experiments.

A hydrated collagen lattice method showed that HSCs underwent significant contraction in the presence of Ang II (*P* < 0.01). The diameters of the gels were measured to evaluate the contraction effect, and we found that carvedilol impeded the gel contraction induced by Ang II in a dose-dependent manner (Figure 4(a)). Under SEM, HSCs in the control group were oval and had long protrusions. Upon Ang II treatment, the morphology of the cells changed rapidly, with the cells extending; carvedilol clearly inhibited this effect (Figure 4(b)). In addition, when the concentration was 15 *μ*M, Y-27632 significantly inhibited Ang II-induced gel contraction (Figure 4(c)).

### 3.4. The Effect of Carvedilol on the AT1R-Mediated RhoA Pathway in HSCs Treated with Ang II In Vitro

**Figure 5:**
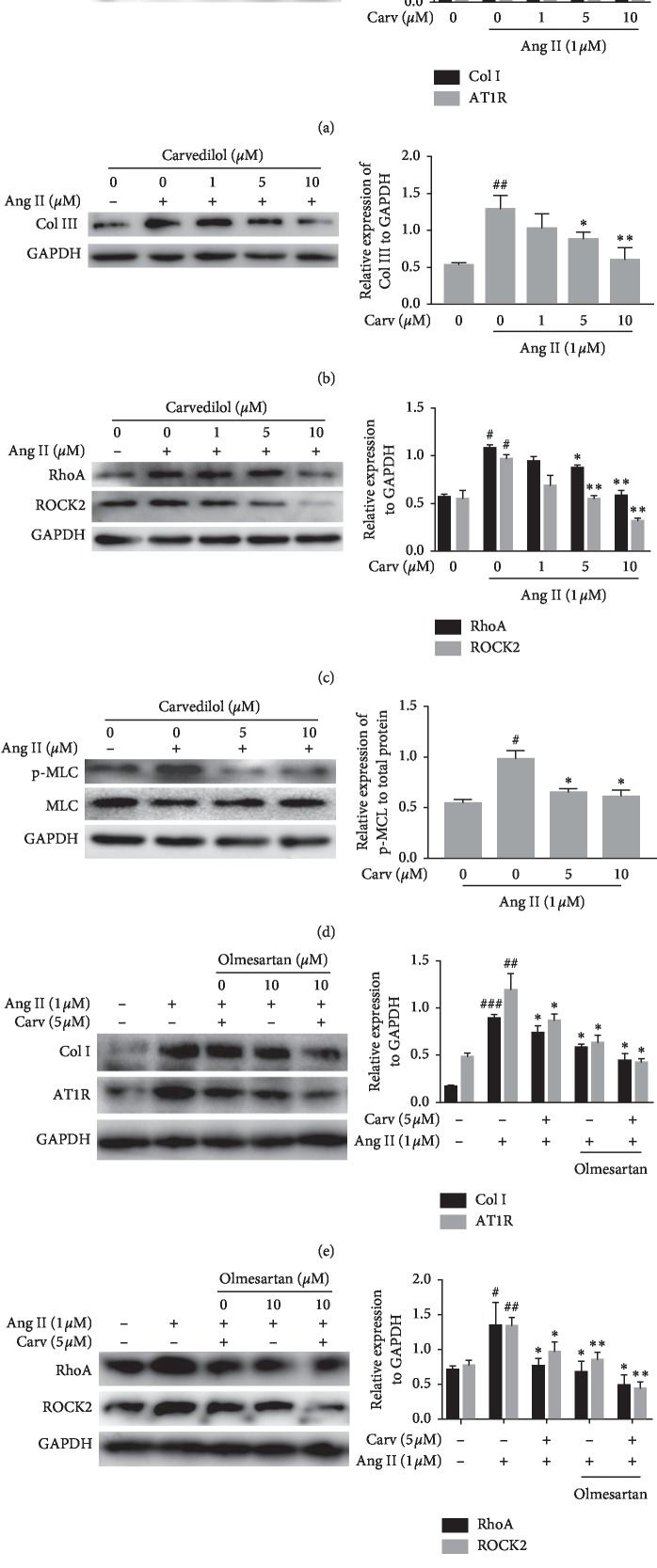
(a–d) HSCs were treated with Ang II (1 *μ*M) and different concentrations of carvedilol. Western blot analyses of collagen I, AT1R, collagen III, RhoA, ROCK2, p-MLC, and MLC. ^#^*P* < 0.05 versus control group; ^##^*P* < 0.01 versus control group; ^*∗*^*P* < 0.05 versus Ang II group; ^*∗∗*^*P* < 0.01 versus Ang II group. (e, f) HSCs were treated with Ang II (1 *μ*M), carvedilol (5 *μ*M), and olmesartan (10 *μ*M). Western blot analyses of collagen I, AT1R, RhoA, and ROCK2. ^#^*P* < 0.05 versus control group; ^##^*P* < 0.01 versus control group; ^###^*P* < 0.001 versus control group; ^*∗*^*P* < 0.05, *P* < 0.05 (Ang II + olmesartan) group versus (Ang II + carvedilol) group, *P* < 0.05 (Ang II + carvedilol + olmesartan) group versus (Ang II + olmesartan) group; ^*∗∗*^*P* < 0.01, *P* < 0.01 (Ang II + olmesartan) group versus (Ang II + carvedilol) group, *P* < 0.01 (Ang II + carvedilol + olmesartan) group versus (Ang II + olmesartan) group. Representative blots were obtained from three independent experiments.

Ang II is considered to play a significant role in liver fibrogenesis by binding to Ang II receptors, which are expressed in activated HSCs. *In vitro* experiments demonstrated that collagen synthesis and cell proliferation were increased in Ang II-treated HSCs and that carvedilol inhibited these effects in a dose-dependent manner (Figures 5(a) and 5(b)). In the Ang II group, the protein expression of AT1R was upregulated compared to that in the control group. After carvedilol treatment, activation and proliferation of HSCs were suppressed, and AT1R expression was also decreased (Figure 5(a)). Moreover, the data showed that activation of the AT1R-mediated RhoA pathway and expression of pathway components were increased in the Ang II group. Additionally, collagen I and III, markers of HSC profibrotic activity, exhibited significantly higher expression in the Ang II group than that in the other groups. However, the expression levels of collagen I, collagen III, RhoA, and ROCK2 were dose-dependently reduced by carvedilol treatment (Figures 5(a)–5(c)). Furthermore, phosphorylation of MCL was decreased with carvedilol treatment such that HSC contraction was inhibited (Figure 5(d)). Olmesartan, an Ang II receptor antagonist, was applied to confirm the role of AT1R blockade in carvedilol inhibition of the RhoA pathway. We found that olmesartan (10 *μ*M) effectively inhibited AT1R protein expression and collagen synthesis (Figure 5(e)). In addition, expression of AT1R-mediated RhoA/ROCK2 pathway components was reduced to a great extent in the olmesartan (10 *µ*M) group, and more significant inhibition of the expression of these proteins was observed after treatment with the combination of olmesartan and carvedilol (Figure 5(f)). The results showed that carvedilol inhibited the expression of AT1R-mediated RhoA/ROCK2 pathway components.

### 3.5. The Effect of Carvedilol on Liver Fibrosis In Vivo

**Figure 6:**
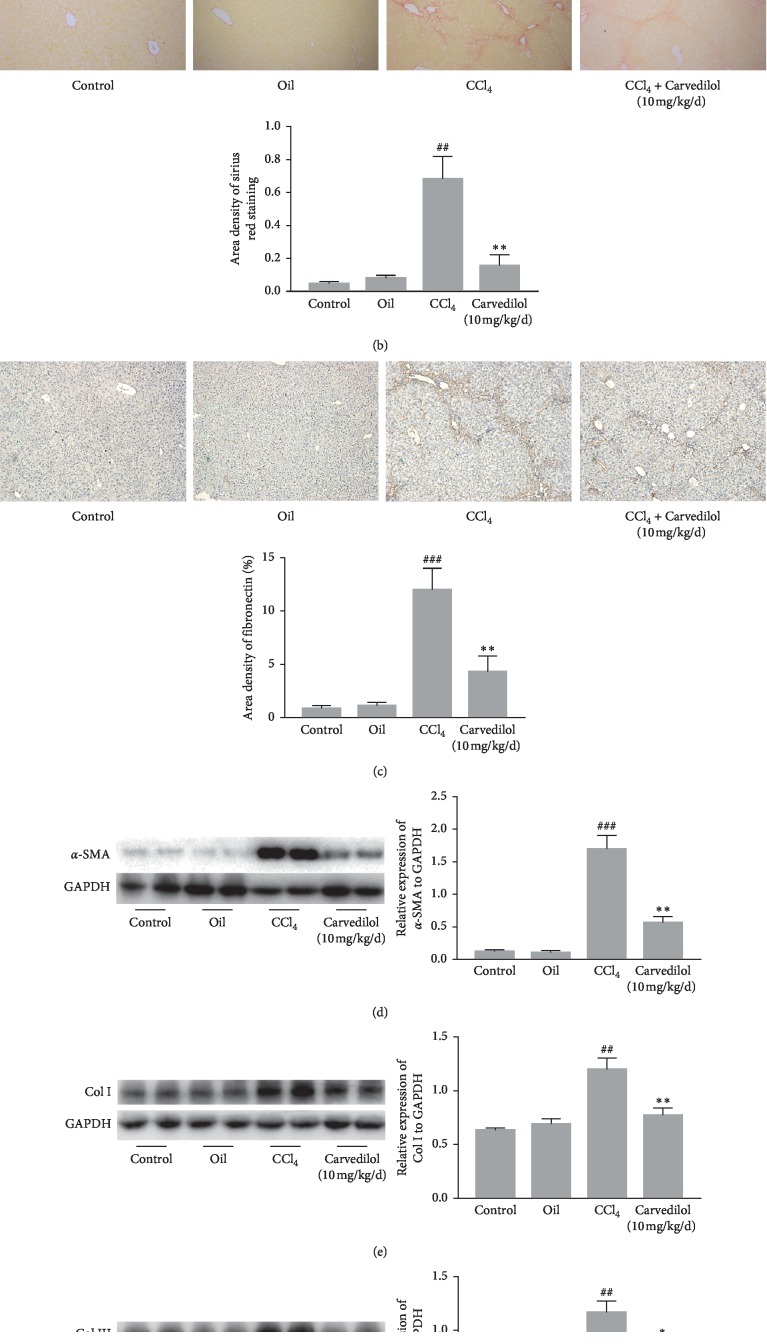
Carvedilol attenuated liver fibrosis in CCl_4_-treated mice. (a) H&E staining (magnification ×100). (b) Sirius Red staining (magnification ×100). Collagen deposition was quantified using Image-Pro Plus software. ^##^*P* < 0.01 versus oil group; ^*∗∗*^*P* < 0.01 versus CCl_4_ group; *n* = 6. (c) The expression of Fibronectin in liver tissues was detected by immunohistochemistry staining (magnification ×100). ^###^*P* < 0.001 versus oil group; ^*∗∗*^*P* < 0.01 versus CCl_4_ group; *n* = 6. (d–f) The expression levels of *α*-SMA, collagen I, and collagen III in liver tissues were detected by Western blot assay. ^###^*P* < 0.001 versus oil group; ^##^*P* < 0.01 versus oil group; ^*∗∗*^*P* < 0.01 versus CCl_4_ group; ^*∗*^*P* < 0.05 versus CCl_4_ group. Representative blots were obtained from three independent experiments.

The effect of carvedilol on liver fibrosis *in vivo* was evaluated by H&E and Sirius Red staining. The liver tissues of mice in the CCl_4_ model group (METAVIR > F2) showed increased inflammatory cell infiltration and fibrous tissue hyperplasia compared to those in the carvedilol treatment group (METAVIR ≤ F2) (Figure 6(a)). Sirius Red staining showed marked collagen deposition in the CCl_4_ model group, whereas the extent of staining was reduced in the carvedilol treatment group (Figure 6(b)). In addition, fibronectin can be used to evaluate the degree of liver fibrosis, and the immunohistochemical staining results showed that carvedilol treatment reduced fibronectin expression in liver tissues (Figure 6(c)). *α*-SMA is a primary fibrotic marker during liver fibrogenesis, and its expression increases after HSC activation. The results showed that the expression of *α*-SMA in the CCl_4_ group was significantly increased but that carvedilol treatment inhibited this CCl_4_-induced increase (Figure 6(d)). Moreover, Western blot analysis showed that the expression of collagen I and III was decreased in the carvedilol treatment group (Figures 6(e) and 6(f)). The data indicate that carvedilol reduced HSC activation and consequently attenuated liver fibrosis.

### 3.6. The Effects of Carvedilol on ACE1 Expression in a Mouse Liver Fibrosis Model

**Figure 7:**
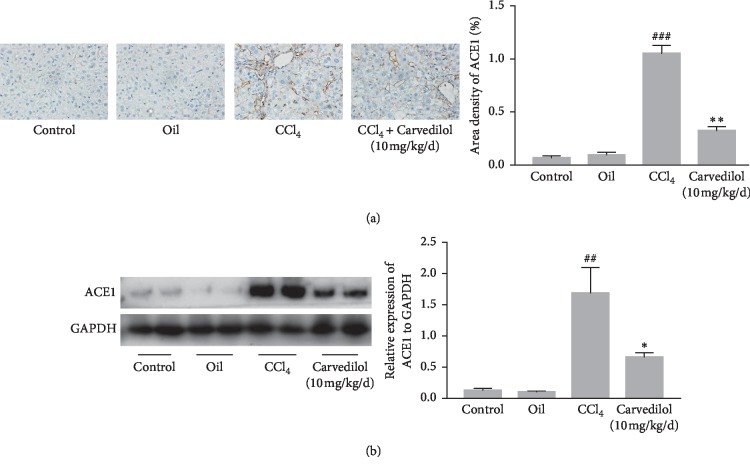
(a) The expression of ACE1 in liver tissues was detected by immunohistochemistry staining (magnification ×500). ^###^*P* < 0.001 versus oil group; ^*∗∗*^*P* < 0.01 versus CCl_4_ group; *n* = 6. (b) The expression level of ACE1 in liver tissues was measured by Western blot assay. ^##^*P* < 0.01 versus oil group; ^*∗*^*P* < 0.05 versus CCl_4_ group. Representative blots were obtained from three independent experiments.

The expression of ACE1 in mice with CCl_4_-induced liver fibrosis was assessed by immunohistochemistry and Western blot analysis (Figures 7(a) and 7(b)). Immunohistochemical staining showed that ACE1 was located primarily in liver vascular endothelial cells and hepatic sinusoidal lining cells. Although ACE1 was barely expressed in the normal control and olive oil groups, its expression was markedly enhanced in liver fibrosis tissues in the CCl_4_ model group. In the carvedilol treatment group, ACE1 decreased with the improvement of liver fibrosis. The results suggest that ACE1 expression is associated with the extent of liver fibrosis. As ACE1 plays a pivotal role in Ang II production, we infer that ACE1 and Ang II are involved in the process of liver fibrogenesis.

### 3.7. The Effect of Carvedilol on the AT1R-Mediated RhoA Pathway In Vivo

**Figure 8:**
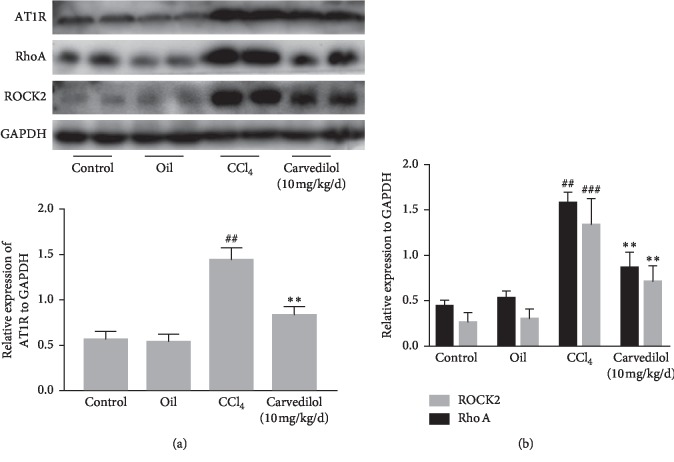
Effect of carvedilol on AT1R-mediated RhoA/ROCK2 pathway *in vivo* was analyzed by Western blot assay. (a, b) Western blot analyses of AT1R, RhoA, and ROCK2. ^##^*P* < 0.01 versus oil group; ^###^*P* < 0.001 versus oil group; ^*∗∗*^*P* < 0.01 versus CCl_4_ group; *n* = 6. Representative blots were obtained from three independent experiments.

Our study further verified the effect of carvedilol on the AT1R-mediated RhoA/ROCK2 pathway in mice with CCl_4_-induced liver fibrosis. The expression and activity of AT1R were upregulated in the CCl_4_ model group, whereas carvedilol treatment attenuated liver fibrosis, leading to a significant decrease in AT1R expression (Figure 8(a)). Additionally, expression of RhoA and ROCK2, downstream effectors of AT1R, was consequently downregulated by carvedilol treatment (Figure 8(b)). These *in vivo* experiments further demonstrate that carvedilol may reduce HSC activation and proliferation, thus inhibiting the AT1R-mediated RhoA pathway.

## 4. Discussion

The development of liver fibrosis and portal hypertension is a multifunctional process involving multiple types of cells, cytokines, chemokines, and growth factors [21]. RAS plays an important role in the development of various chronic liver diseases [22, 23]. Studies have indicated that activated HSCs can express elements of the RAS, including AT1R [24, 25]. Our present research demonstrated that Ang II promoted HSC proliferation, upregulated fibrotic marker expression, and induced cell contraction *in vitro*. However, as a drug commonly used to lower portal hypertension, carvedilol was found to markedly inhibit Ang II-induced effects on cell proliferation and contraction.

HSCs are the major fibrogenic cell type in the liver [26], and the activation, proliferation, and apoptosis of HSCs play crucial roles in liver fibrogenesis. Proliferating HSCs can produce excess ECM components, such as collagen types I and III, which form pathologic fibrous tissues [27]. In our study, a CCK-8 assay demonstrated that carvedilol dose-dependently reduced the proliferation of HSCs induced by Ang II (1 *μ*M). The prolonged G0/G1 phase of the cell cycle showed that carvedilol impeded cell cycle progression in Ang II-treated HSCs, which might be the mechanism by which carvedilol inhibits HSC proliferation. CDK/cyclin complexes play critical roles in cell cycle control: cyclins are considered to aid in the transitions between cell cycle phases, and CDKs drive cell cycle progression [28, 29]. Studies have confirmed that the cyclin D/CDK4 complex controls the G1 phase and that the cyclin E/CDK2 complex can drive the G1/S cell cycle transition [30–32]. In the present study, G1 phase regulatory proteins, including cyclin D, cyclin E, CDK2, and CDK4, were investigated, and the results showed that carvedilol reduced the expression of these regulatory proteins in Ang II-treated HSCs, leading to G1 phase arrest. Furthermore, we observed HSC apoptosis after carvedilol treatment in vitro. Decreased Bcl-2/Bax ratios cause cytochrome C release and trigger caspase cascade activation, resulting in cellular fragmentation [33]. The results showed that carvedilol decreased the Bcl-2/Bax ratio in HSCs, which indicated that mitochondrial apoptosis and HSC proliferation inhibition occurred. In addition, *α*-SMA, collagen I and III are considered markers of HSC activation, proliferation and fibrogenesis, and we found that the expression of *α*-SMA and collagen I and III was downregulated after carvedilol treatment. Both *in vitro* and *in vivo* experiments confirmed that carvedilol inhibited HSC proliferation and collagen deposition, attenuating liver fibrogenesis.

When liver injury occurs, HSCs are activated and become contractile, which increases intrahepatic vascular resistance and plays an important role in the development of portal hypertension [34]. HSC contraction is affected by several vasoactive substances such as Ang II. Studies have shown that hepatic Ang II breakdown and/or angiotensin-(1–7) production can improve intrahepatic resistance [35]. Our *in vitro* study showed that cellular morphology, as observed under SEM, changed when HSC contraction was induced by Ang II and that carvedilol effectively inhibited this response. This finding suggests that carvedilol may further reduce portal hypertension by inhibiting HSC contraction, and this may constitute a molecular mechanism by which carvedilol decreases portal hypertension. Activated HSCs can express AT1R, which in turn plays an important role in HSC activation and fibrogenesis [36–38]. Studies have reported that Ang II receptor antagonists, such as olmesartan, can improve liver fibrosis by suppressing HSC proliferation [7, 8, 22]. The results revealed that carvedilol inhibited HSC activation and proliferation, thus significantly decreasing AT1R expression on the surface of HSCs. On the other hand, the reduction in AT1R expression inhibited collagen synthesis and HSC proliferation. It has been reported that AT1R can stimulate and activate the RhoA/Rho-kinase pathway involved in cell contraction and ECM production [13, 14]. Our *in vitro* experiments demonstrated that the RhoA/ROCK2 pathway was inhibited by carvedilol in a dose-dependent manner and that this effect was associated with AT1R inhibition in Ang II-treated HSCs. The results also suggest that carvedilol may inhibit HSC contraction through the RhoA/ROCK2 pathway. In the gel contraction experiment, Y-27632 could inhibit Ang II-induced HSC contraction, which indicated that ROCK was a downstream effector of carvedilol's effects on HSCs. We also established a mouse liver fibrosis model by intraperitoneally injecting mice with CCl_4_ for 6 weeks, and *in vivo* experiments further verified the effect of carvedilol on the AT1R-mediated RhoA/Rho-kinase pathway, which might affect cell contraction and fibrogenesis.

The RAS plays crucial roles in regulating blood pressure and maintaining electrolyte balance [39]. Inhibition of systemic RAS has been shown to be effective in reducing portal hypertension [40]. In addition to the systemic RAS, studies have indicated that a local RAS exists in multiple organs, including the liver [41, 42]. Ang II is a vasoconstrictor generated by the activity of ACE (ACE1). The intrahepatic RAS is active in chronic liver diseases with increased local levels of Ang II, which can induce an array of fibrogenic actions [38, 43]. Studies have suggested that blocking the local RAS with ACE inhibitors or angiotensin receptor blockers (ARBs) may be effective for antifibrotic therapy [8, 44, 45]. In the present study, ACE expression was significantly higher in liver fibrosis model mice than in control mice. We infer that local production of Ang II may be consequently increased to aggravate liver fibrosis. After carvedilol treatment, the extent of liver fibrosis was attenuated, and the expression of ACE was decreased accordingly. Thus, the results indicate that ACE may be a promising biomarker for liver fibrosis.

## 5. Conclusions

In conclusion, *in vitro* experiments demonstrated that carvedilol inhibited Ang II-induced HSC proliferation, impeded cell cycle progression, and induced HSC apoptosis. *In vivo* experiments further confirmed that carvedilol inhibited HSC activation and proliferation to attenuate liver fibrosis. Carvedilol also influenced elements of the intrahepatic RAS and markedly decreased the expression of ACE in fibrotic liver tissue. Moreover, carvedilol inhibited Ang II-induced HSC contraction by interfering with the AT1R-mediated RhoA/ROCK2 pathway, which may be one of its molecular mechanisms for reducing portal hypertension.

## Data Availability

The data used to support the findings of this study are available from the corresponding author upon request.
